# Perceptions on male involvement in pregnancy and childbirth in Masasi District, Tanzania: a qualitative study

**DOI:** 10.1186/s12978-018-0512-9

**Published:** 2018-04-20

**Authors:** Stephen Oswald Maluka, Apollonia Kasege Peneza

**Affiliations:** 1 Institute of Development Studies, University of Dar es Salaam, P.O. Box 35169 Dar es Salaam, Tanzania; 2 Masasi Town Council, Masasi, Tanzania

**Keywords:** Male involvement, Pregnancy and childbirth, Maternal and child health, Tanzania

## Abstract

**Background:**

Despite the efforts to promote male involvement in maternal and child health, studies in low and middle income countries have reported that male participation is still low. While factors that hinder male partners from participating in maternal and child healthcare are well documented, there is dearth of studies on local perceptions about male involvement in pregnancy and delivery care. The main objective of this study was to explore local perceptions about male involvement in pregnancy and childbirth in Tanzania.

**Methods:**

Semi-structured individual interviews were conducted with key respondents and a thematic approach was used to analyse data.

**Results:**

The findings revealed that women preferred to be accompanied by their partners to the clinics, especially on the first antenatal care visit. Men did not wish to be more actively involved in antenatal care and delivery. Respondents perceived men as being breadwinners and their main role in pregnancy and child birth was to support their partners financially. The key factors which hindered male participation were traditional gender roles at home, fear of HIV testing and unfavourable environment in health facilities.

**Conclusion:**

This study concludes that traditional gender roles and health facility environment presented barriers to male involvement. District health managers should strengthen efforts to improve gender relations, promote men’s understanding of the familial and social roles in reproductive health issues as well as provide male friendly services. However, these efforts need to be supported by women and the society as a whole.

## Plain English summary

Despite the efforts to promote male involvement in maternal and child health, studies in low and middle income countries have reported that male participation is still low. While factors that hinder male partners from participating in maternal and child healthcare are well documented, there is dearth of studies on local perceptions about male involvement in pregnancy and delivery care. The main objective of this study was to explore local ideas about male involvement in pregnancy and childbirth in Tanzania. Semi-structured individual interviews were conducted with key respondents and thematic approach was used to analyse data.

The findings of the study revealed that women preferred to be accompanied by their partners to the clinics, especially on the first antenatal care visit. Men did not wish to be more actively involved in antenatal care and delivery. The society perceived men as being breadwinners and their main role in pregnancy and child birth was primarily to support their partners financially. The key factors which hindered male participation were traditional gender roles at home, fear of HIV testing and unfavourable infrastructure in health facilities.

This study concludes that traditional gender roles and health facility environment presented barriers to male involvement during antenatal care and delivery. The study recommends that district health managers should strengthen efforts to improve gender relations, promote men’s understanding of the familial and social roles in reproductive health issues as well as provide male friendly services. However, these efforts need to be supported by women and the society as a whole.

## Background

Until recently, pregnancy and childbirth matters had to a large extent been viewed as the domain of women while men remained at the periphery [1]. Men were mainly responsible for providing money for medical bills and other material needs as well as naming the new born [2, 3].

Since the mid 1990s there has been a growing impetus to involve men in pregnancy and child birth programmes as partners, fathers, community members, and key decision-makers of the households [4]. For example, in the United Kingdom (UK), men have been actively included in maternal and child health programmes since the 1970s [5]. In Sweden and Norway, men’s participation in maternal and child health has for a long time been emphasised in legislation [6, 7].

In many African countries, the interest for male involvement has been largely driven by the need for HIV testing as part of the prevention of mother-to-child transmission of HIV (PMTCT). Studies have reported that participation of male partners in antenatal care (ANC) counselling can considerably increase women’s utilisation of HIV-related services [8–12]. Conversely, low participation of men can negatively impact on access to and utilisation of maternal and child health services, including HIV testing and PMTCT [13–15]. Family planning has also been reported to improve when men are included in the counselling sessions with their wives [16, 17]. Furthermore, other studies have shown that male involvement results in the increased access to postpartum services [18]; reduced maternal smoking and depression; and reduced risks of infant mortality [18–21].

However, other studies have reported negative and unintended consequences of male involvement in ANC programmes and interventions [22–24]. For instance, a study on counselling for HIV testing in Tanzania revealed that some pregnant women did not return for the second visit when requested to come back for ANC services together with their male partners [23]. Another study also indicated that utilisation of PMTCT services decreased when unmarried women were required to come with their partners [24]. Further to that, a recent study in Malawi reported several unintended consequences of male involvement in maternal and nutrition programmes, including discrimination against women, marginalisation of married women and reinforcing men’s decision-making roles [25].

In Tanzania, male involvement in maternal and child health care related matters is relatively new. Pregnancy and childbirth have traditionally been considered as women’s affairs and pregnant women were given support by their mother-in-laws, sisters, and other women relatives during labour and birth [26].

As a result of global interests in male involvement in improving access to and utilisation of the maternal, new-born and child health (MNCH) services, the Tanzania government’s maternal and child health policy documents describe the need for male involvement [27]. Likewise, as part of the antenatal care policy, couples are encouraged to attend clinics together [28]. During ANC visits, health workers provide health education on danger signs during pregnancy, the general care of the pregnant woman at home, and discuss birth preparedness (BP) and complication readiness (CR) plans [28]. In addition, a key component of the national policy on PMTCT is male involvement in HIV counselling and testing (HCT) [28].

However, research in low and middle-income countries have reported low male participation in maternal and child health matters [10, 12–14, 29–31]. Similarly, several studies conducted in Tanzania have reported low rate of male involvement in the PMTCT as well as in HCT [11, 13, 14, 30, 31].

While factors that hinder male partners from participating in maternal and child healthcare are well documented [10–14, 32], there is dearth of studies on local perceptions about male involvement in pregnancy and delivery care. This paper describes perceptions about male involvement in pregnancy and childbirth in Masasi District in Tanzania.

## Methods

### Study settings

The study was conducted in Masasi District Council in Mtwara Region in Southern Tanzania. Masasi District is natively inhabited by Makua, Yao, Mwera and Makonde communities. The District is predominantly rural and the majority of the population rely on agriculture for subsistence and production.

### Study design and data collection

We conducted a cross sectional qualitative case study. The study relied on semi-structured interviews with key respondents. Masasi District was purposively selected because the first author had an active role in the district. Data were collected by a trained qualitative research assistant from April to June 2016. Purposive sampling technique was used to recruit all the interviewees. Interviews were conducted with health care providers, social workers, community and village leaders, and traditional birth attendants. Leaders and traditional birth attendants were recruited in their respective villages surrounding the health facilities. Interviews were also carried out with pregnant women who were attending ANC clinics and women who had delivered in the last 12 months. In addition, male partners who had accompanied their wives for ANC, delivery and postnatal care services were also interviewed. Women and male partners were recruited from six randomly selected health facilities: the district hospital, one health centre, and four dispensaries. Interviews were carried out in a private room at the respective health facilities where women and male partners were located. In order to avoid male partner’s dominance and bias, couples were interviewed separately. Discussion guides were developed for each category of respondents. Interviews lasted between 20 and 30 min and were audio-recorded with permission of the respondents. As indicated in Table 1, a total of 53 interviews were carried out.

**Table 1:** Category of respondents

S/N	Category of respondent	Number
1	Pregnant women	8
2	Women who had delivered within the last 12 months	12
3	Male partners	13
4	Health providers	6
5	Traditional birth attendants	3
6	Religious leaders	5
7	Village leaders	5
8	District Health Managers	1
Total		53

### Data processing and analysis

The study used a thematic approach to analyse the data [33]. First, recorded interviews were transcribed verbatim in Kiswahili by the first author (AKP) and checked for accuracy by the second author (SOM). Second, AKP developed a codebook based on the objectives of the study, and AKP coded the data manually based on a predefined codebook. SOM checked for accuracy during the coding process. Additional codes which emerged during coding were added concurrently following consensus of both authors. Saturation was achieved when no more codes emerged from the data. After this process, we sorted and grouped the data under patterns that were considered to be more generalisable. Finally, summaries and syntheses were generated and key terms, phrases and expressions of the respondents were used to support the findings.

## Results

The findings of the study have been presented in three broad themes, namely traditional gender roles at home, fear of HIV testing, and unfavourable infrastructure in health facilities.

### Traditional gender roles at home

There were mixed perceptions among women and community leaders regarding men accompanying their partners to ANC clinics. Female respondents preferred to be accompanied by their partners during the first ANC visit to receive HIV testing. Similarly, male respondents reported that they attended the first ANC visits in order to test for HIV. They also attended ANC to ensure that their partners are received at the ANC clinic by the health providers. One respondent had this sentiment:

*It is important for the couple to undergo medical check-up so that we can get treated in time if we happen to have medical problems to avoid infecting the child* (Pregnant woman).

Another responded said:

*“It is important to accompany a wife as she attends clinics because there at the clinics, there are instructions that we are given by nurses which the father also needs to know. It is also important for both of you to get tested to avoid infecting the baby if we are infected” (A male partner).*

However, while a few female respondents preferred to be accompanied by their male partners during routine ANC visits, some women and community leaders did not see the importance of men attending routine ANC visits.

*“I am happy to go to the clinic alone. I only need my husband to attend the first antenatal visit for HIV testing”* (A pregnant woman).

Some female respondents did not see any problem to attend routine ANC visits without their partners. One respondent exemplified this way:

*“For routine visits, I continued coming alone as I did not see any justification for my husband to come. I thought it better for him to continue with other duties. I just ask him to hire a motorcycle for me”* (A Woman with a child under 12 months).

Another respondent added this way:

*“For routine clinics, I come alone. I have never asked my husband to accompany me to the clinics on routine visits”* (A pregnant woman).

There was consensus among all types of respondents that men were the main breadwinners of the households. Men were supposed to support their partners financially and they were in charge of preparing for delivery. In particular, men had the responsibilities of preparing essential items required for delivery like gloves, clothes (khangas or vitenge), makintosh, a basin or a bucket, razor blades and money in case of emergencies.

All categories of respondents reported that the gender roles in the community were strengthened by the tradition of *jando* and *unyago* which was still practiced in the study area. According to the tradition, at the age of puberty boys and girls are taken separately to the bush or any appropriate place and trained on their responsibilities as mothers and fathers. This tradition puts clear a division of responsibilities between boys and girls. Among other things, boys are told not to get involved in women’s activities and the vice versa. According to this tradition, issues related to ANC, pregnancy care and childbirth were defined as women affairs. Attending clinics for example, was categorized as solely women’s role. A man who was seen to attend clinics with his wife was perceived to be under the control of a woman, which was seen as shameful for men. One respondent narrated this way:

*“One day my friend went to the clinic with her wife. After coming back as we were chatting, I asked him where he had come from. He said he had taken his wife to the clinic. Other friends intervened” “You are hopeless, what has the woman done to you?...”* (A male partner with a pregnant woman).

As for participation of men during delivery, generally, men did not prefer to be in the delivery room with their partners. Men preferred their spouse to be accompanied by their mother in-law, sisters, and other female relatives. One respondent clarified this position:

“*Some of the women use abusive language because of the pain they get during the time of labour. It is not good for men to be in the labour room”* (A male respondent).

Another respondent added:

*“Sometimes the complaints of a woman due to labour pains are full of shameful statements for the man to hear”* (A male respondent).

A similar view was shared by the female respondents, community leaders, and health providers.

*“It is not important for the father to accompany the mother on the delivery day because that is the duty of the women. Even if he goes there, he remains outside”* (A female respondent).

When asked to mention people who would like to accompany them during delivery, female respondents mentioned female relatives such as mothers, aunts, grandmothers, sisters, and sisters-in-law. Most often, men featured last in the list and their roles were mainly to pay for medical bills and be responsible in case of emergencies and referrals.

There was systematic distribution of responsibilities between male and female relatives in handling a woman during labour and delivery. Women relatives, such as mothers, grandmothers, sisters, sisters-in-law and traditional birth attendants (TBAs) were responsible for accompanying a pregnant woman to the health facility for delivery. TBAs were found to be important in case a woman gave birth before reaching the health facility. Other relatives such as mothers, grandmothers, sisters, and sisters-in-law were found to be important in taking care of a pregnant woman and newly born child while at the health facility. Female relatives were responsible for preparing food and washing clothes.

Men’s responsibilities included the preparation of means of transport to and from the health facility, purchasing essential delivery items and preparing money for meeting the living expenses and other requirements at the health facility as might be recommended by the health workers.

### Fear of HIV testing

With regards to antenatal care attendance, respondents reported that pregnant women and their spouses who were not sure of their HIV status were afraid of going together for ANC services. It was revealed by different respondents that one of the basic services provided at the clinic during the first ANC visit was HIV counselling and testing. According to our respondents, the majority of men did not want to take HIV testing. To them, testing for HIV was soliciting problems. One respondent explained:

*Husbands’ response for accompanying their wives as they attend clinics is low because they are afraid of HIV testing. If you ask them as to why they do not want to get tested for HIV, they say testing for HIV is soliciting problems. It is better not to know their HIV status* (Male village government leader).

### Unfavourable environment in health facilities

Respondents reported that the infrastructure in most of the health facilities was not appropriate for men. According to our respondents, in almost all health facilities, several women could be in a labour room at the same time, with no privacy and thus it was not suitable for a man to be in the room and watch someone’s wife to deliver. Therefore, due to lack of space in the delivery rooms, men were not allowed.

*“As far as our hospital environment is concerned, men are not allowed in the delivery room. Normally, men stay outside”* (Male partner).

Another respondent added:

*“A husband is not allowed to get in. It is only female relatives that are allowed to get in. We do not allow men because the labour room is only one where you can find more than one woman. So we cannot allow men for privacy”* (Health worker).

However, when male respondents were asked whether they would be present during delivery if there was a change in infrastructure to improve privacy, there were mixed responses. While a few male respondents agreed to be with their partners in the delivery room, others pointed out that their culture does not support men watching pregnant women during delivery. They pointed out that in most cases, during delivery pregnant women use shameful statements which are not morally good for men to hear.

## Discussion

The findings of this study revealed that men did not wish to be more actively involved in ANC and delivery. Respondents perceived men as being breadwinners and were not only supposed to support their partners financially, but also be in charge of preparing for delivery. In particular, men had the responsibilities of preparing essential items required for delivery and facilitating women to reach health facilities. Similarly, women were happy to go to the clinics alone, but they wanted men to go for the first antenatal visit for HIV testing. The findings of this study corroborates previous studies that suggest that traditional gender norms continue to impede men’s participation in pregnancy and childbirth related services [2, 10–15, 25].

Given the importance of ANC, facility delivery and postpartum care, active involvement of men is important. ANC visits are opportune moments for couples to receive health information about risks during pregnancy and childbirth [28]. The couples also receive important information to enable them to better prepare for the facility delivery [28]. The district health managers and other stakeholders involved in maternal and child health should strengthen efforts to improve gender relations, promote men’s understanding of the familial and social roles in reproductive health issues as well as provide male friendly services.

Some possible ways of engaging men could be through working with male champions and male gate keepers. Male champions could be identified in the communities and capacitated to educate other men on the importance of participating actively in maternal and child health care. In addition, male gate keepers such as community and religious leaders should be used to promote male involvement in maternal and child health. Interventions that promote gender equality and treat men as agents of change have been reported to improve communication for couples, reduce maternal workload and increase involvement of male partners in birth preparedness [1, 34, 35].

While we recognise the importance of couple counselling during ANC visits, it is important to create separate activities for men and women during ANC visits [25]. This means that programmes that are designed to engage men in maternal and child health should provide a safe space for couples to engage but also provide separate spaces for each of the sexes to express themselves [25].

In addition to the ANC attendance, community health workers (CHW) could be capacitated to conduct home-based visits and provide training to the members of the community on danger signs during pregnancy, birth preparedness arrangements, complication readiness, and promote health seeking behaviours. A recent study in Tanzania showed that training provided by the CHW to the community members increased men’s knowledge of maternal healthcare, improved joint decision-making, and increased attendance of spouses in ANC and delivery [36].

The findings of the study further reported that fear of HIV testing discouraged men to accompany their partners to health facilities for ANC. Earlier studies have reported that the vast majority of men fear to receive HIV positive results. This is due to the HIV-associated stigma and disclosure [15, 37–39]. While studies have reported that with the availability of life-prolonging antiretroviral therapy, people tend to accept HIV status and that HIV is now becoming accepted in the society [37, 39], perceived stigma seems to continue influencing individuals’ decisions to disclose or not to disclose their HIV status ([37–44]). It is also possible that men fear testing for HIV positive in the presence of their partners because of the implications on their marriage or relationships. There is need of further sensitizing men and the community at large on the importance of testing for HIV during routine ANC visits. Kululanga and colleagues [42] have shown how the use of incentives, male peer initiatives and sensitisation campaigns motivated men to participate in antenatal clinics in Malawi. Therefore, the efforts done by the government and other stakeholders to address the stigma related to the HIV disclosure should be continued.

The study revealed that health facilities had unfavorable environment for men’s participation during delivery. For example, the physical infrastructures hampered male participation in maternal and child health. It was evident from the findings that due to the nature of delivery rooms, men were not allowed in the labour room. The health system attempts to protect the privacy of other women because the delivery room is structured in a way that contains many delivery beds and there are always other women in the room. Lack of space to accommodate male partners during delivery has been reported to deter participation of men in pregnancy and child birth [10, 32, 43, 44]. This finding implies that interventions to promote male involvement in reproductive and maternal health care services require a major transformation of the health system physical infrastructures to create environments that would allow for male participation in antenatal, delivery as well as postnatal care services.

### Limitations of the study

First, this study was conducted in health facilities. Therefore, only men who had accompanied their partners to ANC were interviewed. It is possible that interviews with men who had not accompanied their partners would provide additional and/or different information on men’s involvement in maternal and child health. Second, the study was conducted in only 6 health facilities in one district. The findings may not be generalised to other districts in Tanzania as the infrastructure across other districts may be different. Third, the majority of interviewees in this study were female. Hence, the findings may reflect more female’s opinions. Furthermore, the study was conducted in a rural district. The perceptions on male involvement may be different if the study was conducted in urban settings where normally the infrastructure in health facilities is relatively better. Notwithstanding these limitations, this study provides useful insights on local perceptions and factors that hinder male involvement in pregnancy and delivery care in Tanzania.

## Conclusion

This study aimed at exploring local perceptions about male involvement in pregnancy and delivery care. The study concludes that traditional gender roles, HIV testing policy and health facility environment presented barriers to male involvement in pregnancy and childbirth. The findings suggest that the district health managers and other stakeholders involved in maternal and child health should strengthen efforts to improve gender relations, promote men’s understanding of the familial and social roles in reproductive health issues as well as provide male friendly services. Furthermore, health promotion campaigns should be strengthened in the communities. The campaigns should focus on safer conception and options for discordant couples. These may improve awareness and could assist in eliminating a bit of fear from the HIV testing process if couples know that they still have options as discordant couples. However, these efforts need the support of women and the society as a whole.

## Abbreviations

AIDS: Acquired Immune Deficiency Syndrome
ANC: Antenatal care
HIV: Human Immunodeficiency Virus
ICPD: International Conference on Population and Development (ICPD)
MNCH: Maternal, new-born and child health
NGO: Non-Governmental Organization
PMTCT: Prevention of mother to child transmission
PNC: Postnatal care
RCH: Reproductive and child health
VCT: Voluntary counselling and testing
WHO: World Health Organization
